# Control of Viremia Enables Acquisition of Resting Memory B Cells with Age and Normalization of Activated B Cell Phenotypes in HIV-Infected Children

**DOI:** 10.4049/jimmunol.1500491

**Published:** 2015-06-26

**Authors:** Daniel M. Muema, Gladys N. Macharia, Amin S. Hassan, Shalton M. Mwaringa, Greg W. Fegan, James A. Berkley, Eunice W. Nduati, Britta C. Urban

**Affiliations:** * Kenya Medical Research Institute-Wellcome Trust Research Programme, Centre for Geographic Research - Coast, 80108 Kilifi, Kenya;; † Centre for Clinical Vaccinology and Tropical Medicine, University of Oxford, Oxford OX3 7LJ, United Kingdom; and; ‡ Department of Parasitology, Liverpool School of Tropical Medicine, Liverpool L3 5QA, United Kingdom

## Abstract

HIV affects the function of all lymphocyte populations, including B cells. Phenotypic and functional defects of B cells in HIV-infected adults have been well characterized, but defects in children have not been studied to the same extent. We determined the proportion of B cell subsets and frequencies of Ag-specific memory B cells in peripheral blood from HIV-infected children and healthy controls, using flow cytometry and B cell ELISPOT, respectively. In addition, we measured the quantities and avidities of plasma Abs against various Ags by ELISA. We also determined plasma levels of BAFF and expression of BAFF receptors on B cells. Children with high HIV viremia had increased proportions of activated mature B cells, tissue-like memory B cells and plasmablasts, and low proportions of naive B cells when compared with community controls and children with low HIV viremia, similar to adults infected with HIV. HIV-infected groups had lower proportions of resting memory B cells than did community controls. Notably, high HIV viremia prevented the age-dependent accumulation of class-switched resting memory B cells. HIV-infected children, regardless of the level of viremia, showed lower quantities and avidities of IgG and lower frequencies of memory B cells against Expanded Program on Immunization vaccines. The HIV-infected children had an altered BAFF profile that could have affected their B cell compartment. Therefore, B cell defects in HIV-infected children are similar to those seen in HIV-infected adults. However, control of HIV viremia is associated with normalization of activated B cell subsets and allows age-dependent accumulation of resting memory B cells.

## Introduction

Although HIV primarily targets the CD4^+^ T cell compartment, it also affects other lymphocyte populations, including B cells. It causes generalized activation of B cells, as characterized by hypergammaglobulinemia (1), increased production of autoantibodies (2), increased susceptibility to B cell lymphomas (3), expansion of B cell areas in lymphoid tissues (4), spontaneous in vitro production of Igs by PBMCs, overexpression of markers of activation, and terminal differentiation of B cells (5, 6). Yet, HIV-viremic patients have poor Ab responses to vaccines, and their B cells are refractory to conventional B cell stimulants in vitro (5).

In adults, the dysregulation of B cells by HIV has been well studied. Viremic HIV-infected adults have increased proportions of activated mature B cells, plasmablasts, immature/transitional B cells, and tissue-like memory B cells (7, 8). The proportions of resting memory B cells are reduced, suggesting that HIV depletes B cell memory, as supported by the observed low frequencies of vaccine-specific memory B cells and reduced levels of anti-vaccine plasma Abs (7–9). In addition, HIV in adults is associated with increased plasma concentrations of BAFF and altered expression of BAFF receptors on B cells (10, 11).

Control of viremia with highly active antiretroviral therapy (HAART) resolves most of the derangement in B cell subset distribution and function, but the proportion of resting memory B cells remains relatively lower than that of individuals uninfected by HIV. Ag-specific Ab levels and memory B cells do not spontaneously recover upon control of viremia, suggesting that the depletion of B cell memory is not immediately reversible (8).

In children vertically infected with HIV, immunity to pathogens develops in the presence of HIV, and its effect is likely to be more severe, resulting in low or no acquisition of immunity. Given that protection by Expanded Program on Immunization vaccines and immunity to common childhood pathogens often correlate with humoral responses, we investigated whether HIV-infected children developed memory B cells to the same extent as uninfected children or whether they showed the same defects in their B cell compartment as HIV-infected adults.

## Materials and Methods

### Study population and recruitment

At the time of this study, children born of HIV-infected mothers were registered in the Comprehensive Care and Research Centre at the Kilifi County Hospital. From 2010, HIV-infected children were treated in accordance with the World Health Organization (WHO) guidelines: all those <24 mo: HAART; 25–59 mo: HAART if CD4^+^ T cell percentage <25% and/or if in WHO clinical stage 3 or 4; >60 mo: HAART if their CD4^+^ T cell percentage <20% and/or if in WHO clinical stage 3 or 4 (12). Clinical, laboratory and demographic data were available through the clinical database.

HIV-infected children aged 18 mo–10 y were recruited between October 2010 and May 2012 if it was their first visit to the Comprehensive Care and Research Centre clinic or if they had received cotrimoxazole prophylaxis for ≥6 mo or if they had received both cotrimoxazole and HAART for ≥6 mo. Healthy age-matched children were recruited from the community. All children provided a 5-ml venous blood sample upon recruitment. Information on vaccination status was collected at the time of recruitment. Approval for the study was given by the Kenyan National Ethical Review Committee (SSC No. 1633 and SCC No. 1131). Written informed consent was obtained from each participant’s parent or guardian.

### Viral load determination

HIV viral loads were determined at the International Centre for Reproductive Health, Mombasa, Kenya, using a generic HIV viral load assay (Biocentric): an RT–quantitative PCR test developed by the Agence Nationale de Recherches sur le SIDA, targeting a well-conserved LTR region with a detection limit of 300 RNA copies per milliliter (13).

### B cell subset determination in whole blood by multiparametric flow cytometry

The following Abs were used: anti-CD19–ECD, anti-CD27–Pe-Cy5, anti-CD10–FITC, anti-IgD–PE (Beckman Coulter); anti-CD20–APC-H7, anti-CD21–APC (BD Biosciences); anti-CD21–PE, anti-CD38–Pe-Cy7, anti-BR3–FITC (eBioscience), and anti–transmembrane activator and calcium modulator and cyclophilin ligand interactor (TACI)–PE (R&D Systems).

Whole blood was washed and stained with cocktails comprising the above Abs, and the RBCs were lysed. At least 80,000 PBMCs were acquired on a nine-color CyAn ADP flow cytometer (Beckman Coulter). The data were analyzed using FlowJo software version 9.2 (TreeStar, FlowJo Africa scheme).

The following B cell subsets were identified based on indicated surface markers: immature/transitional, CD19^+^CD10^+^CD38^++^CD27^−^; naive, CD19^+^CD27^−^CD21^+^; tissue-like memory, CD19^+^CD27^−^CD21^−^; resting memory, CD19^+^CD27^+^CD21^+^; activated mature, CD19^+^CD27^+^CD21^−^; plasmablasts, CD19^+^CD27^++^CD38^+++^; unswitched resting memory, CD19^+^CD21^+^CD27^+^IgD^+^; and switched resting memory, CD19^+^CD21^+^CD27^+^IgD^−^. The expressions of BR3 and TACI were also evaluated. The gating strategy is shown in Supplemental Fig. 1.

### Determination of quantities and avidities of Abs to representative vaccines and common childhood infections

Plasma samples were tested for IgG, using a previously established ELISA protocol with various modifications (14). Plates were coated with tetanus toxoid (TT) (Statens) at 1 μg/ml, measles Ag (Meridian) at 5 μg/ml, or a mixture of pneumococcal capsular polysaccharides (serotypes 1, 5, 6B, 14, 19F, and 23F; American Type Culture Collection), each at 10 μg/ml. Plasma samples were diluted at 1:200. HRP-conjugated donkey anti-human IgG diluted at 1:5000 was used as the secondary Ab, whereas OPD substrate (Sigma-Aldrich) was used in accordance with the manufacturer’s instructions. Ab concentrations were expressed as arbitrary units calculated against the plate-specific standard curve generated using a plasma sample that was reactive to the Ag.

For determination of the avidity of IgG Abs, the avidity index was calculated as the ratio of the quantity of Abs in guanidine hydrochloride–eluted wells to the quantity in the control wells (15).

### Determination of frequencies of Ag-specific memory B cells by cultured B cell ELISPOT

Cultured B cell ELISPOTs were done using a method published by Crotty et al. (16), with some modifications. A mixture of stimulants containing 2.5 μg/ml CpG ODN 2006 (Hycult), 1:5000 dilution of *Staphylococcus aureus* Cowan Strain protein A (Sigma-Aldrich), and 0.083 μg/ml pokeweed mitogen (Sigma-Aldrich) was used to stimulate 2 × 10^5^ PBMCs in U-bottomed 96-well plates for 6 d.

Multiscreen plates (Millipore) were precoated with TT (5 μg/ml), measles Ag (5 μg/ml), a mixture of polysaccharides (serotypes 1, 5, 6B, 14, 19F, and 23F, each at 10 μg/ml conjugated with 10 μg/ml methylated human serum albumin) (17), goat antihuman IgG (10μg/ml) or 1% BSA (nonspecific protein control).

Cultured PBMCs were seeded onto previously blocked plates at either 200 or 2000 cells per well (total IgG responses) or at 2 × 20^5^ cells per well (Ag-specific responses) and then incubated overnight. Alkaline phosphatase–conjugated donkey anti-human IgG (Jackson) diluted with 10% New Borne Bovine Serum/PBS at 1:1000 was used as the secondary Ab. Spots were developed using alkaline phosphatase conjugate substrate (Bio-Rad). The plates were later scanned and spots counted using ImmunoCapture software version 6.4 and ImmunoSpot version 5.0 software on a CTL Immunospot analyzer, respectively.

### Statistical analysis

The data were analyzed using STATA version 13.1 (STATA). Those *p* values < 0.05 were considered significant.

Because viremia has been shown to be a major determinant of B cell defects in adults (18), HIV-infected children were stratified into two groups (high- and low-viremia groups) based on a cut-off of 5000 RNA copies per milliliter, the WHO definition of virological failure. Comparisons between groups were done using the Wilcoxon rank sum test (Mann–Whitney *U* test). Because the data were skewed, quantile regression models were used in multivariable analyses. Plotted predicted values showing statistical interaction were obtained from quantile regression models using the PREDXCON Stata add-on package (19). Correlation analyses were done using the Spearman rank-order correlation. Bonferroni correction for multiple comparisons was applied to the correlation analyses.

## Results

### Changes in B cell subset distribution in children infected with HIV

In the first phase of the study, the distributions of B cell subsets were analyzed in 78 HIV-infected children, of whom 36 had high viremia and 42 had low viremia, and in 28 healthy community controls (Table I).

**Table I.** Baseline characteristics of the study participants whose samples were analyzed for B cell subset distribution

	High Viremia		Low Viremia		Community Controls
	Value	*p* Value*^a^*	Value	*p* Value*^a^*	
*n*	36		42		28
Age (y)	3.6 (2.6–5.0)	0.0503	3.8 (2.1–6.7)	0.1861	4.6 (3.2–7.2)
% Female (*n*)	47 (17)	0.614	57 (41)	0.683	54 (15)
% on HAART (*n*)	31 (11)	N/A	69 (29)	N/A	N/A
Viral load*^b^*	4.8 (4.4–5.3)	N/A	1.3 (0.0–3.2)	N/A	N/A
% CD4^+^ T cells*^c^*	22.2 (9.2–26.8)	<0.0005	29.4 (22.5–34.6)	0.0102	33.8 (28.1–39.3)
% CD8^+^ T cells*^c^*	37.1 (31.5–44.9)	<0.0005	27.0 (22.2–39.3)	<0.0005	15.8 (13.7–17.0)
% B cells*^c^*	17.8 (9.8–22.2)	0.061	15.3 (11.2–20.7)	0.009	20.5 (15.7–23.7)

Values shown are medians, 25th and 75th percentiles, unless otherwise stated. Statistical test: Wilcoxon rank sum test, except for % female, where χ^2^ test was used.

^*a*^ The *p* values correspond to comparison between the community controls and either high viremia or low viremia HIV-infected groups.

^*b*^ Viral load is in log_10_ RNA copies per milliliter.

^*c*^ Percentage of total lymphocytes.

N/A, not applicable.

**FIGURE 1.**
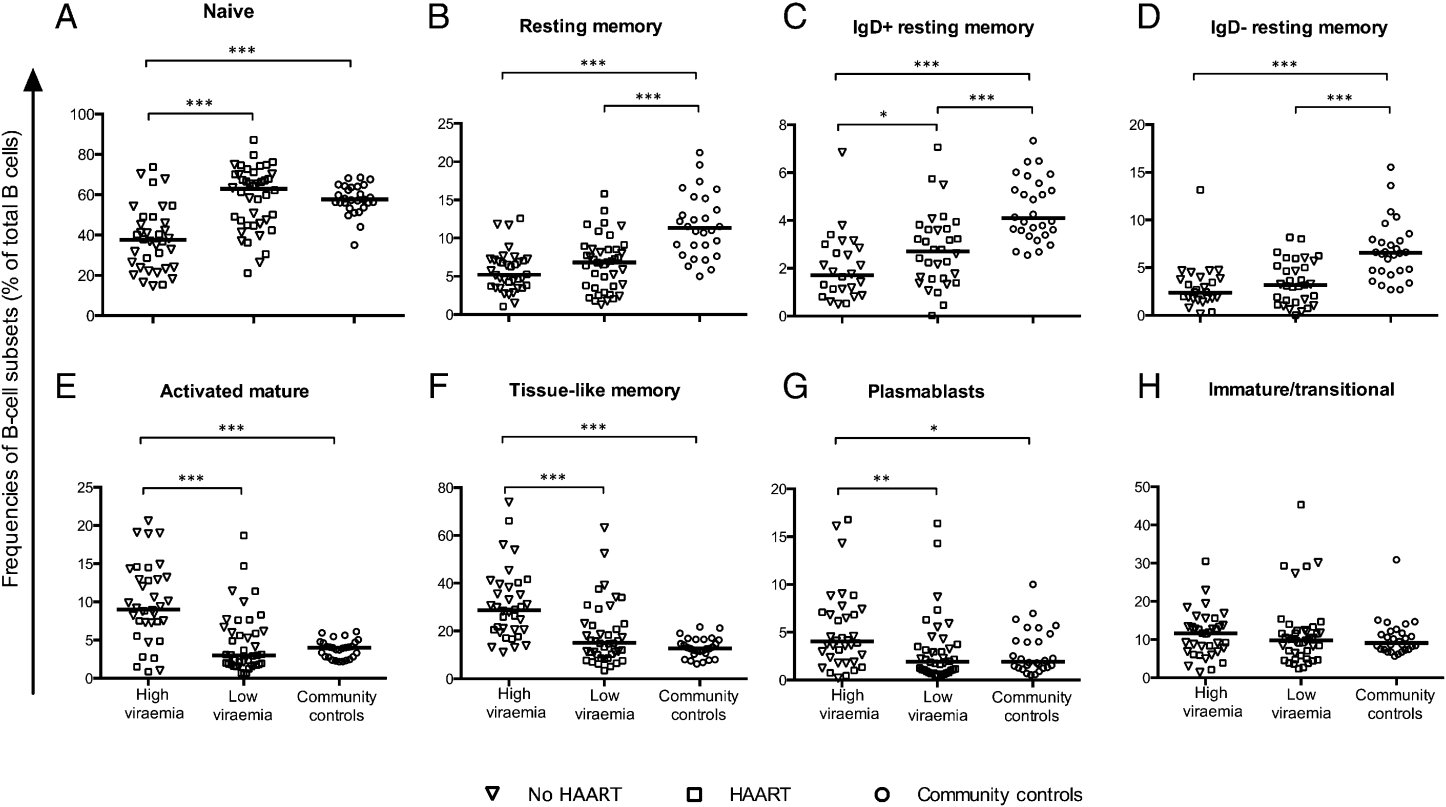
High HIV viremia in children is associated with low frequencies of memory B cells and expansion of activated B cell subsets (activated mature B cells, tissue-like memory B cells, and plasmablasts). (**A**–**H**) Comparison of the proportions of different B cell subsets (as percentage of total B cells) between the study groups after stratifying the HIV-infected children on the basis of their level of viremia. Each symbol represents an individual child. Horizontal lines represent medians. High-viremia group, ≥5000 RNA copies per milliliter; low-viremia group, <5000 RNA copies per milliliter. Wilcoxon rank-sum test. *p < 0.05, **p < 0.01, ***p < 0.001.

The proportions of naive B cells were significantly lower in the high-viremia group than in both the low-viremia group and the community controls. Both the high- and low-viremia groups had lower proportions of total, switched, and unswitched resting memory B cells than did community controls. The proportions of activated mature B cells, tissue-like memory B cells, and plasmablasts were significantly increased in the high-viremia group, but not in the low-viremia group, when compared with community controls (Fig. 1).

Among the 78 HIV-infected children, 40 were receiving HAART, enabling analyses on the effect of HAART within each group defined by viremia. There were no major differences in characteristics between children receiving HAART and those who did not in either group, other than age. In the low-viremia group, HAART-naive children tended to be younger than children receiving HAART, suggesting that low viremia in the absence of HAART was an indication of early HIV as opposed to a special ability to control viremia (Supplemental Table I). In the high-viremia group, HAART-treated children had lower proportions of activated mature B cells than did HAART-naive children (*p* = 0.0067), most likely because treatment failure was a recent event or HAART reduced B cell activation independent of viremia. In the low-viremia group, proportions of tissue-like memory B cells were higher in HAART-naive children than in HAART-treated children (*p* = 0.0128). Adjusting for multiple comparisons did not change the interpretation of the data on distribution of subsets of B cells, with the exception of plasmablasts, for which the difference between highly viremic children and community controls became statistically insignificant.

### Control of viremia results in age-appropriate acquisition of memory B cells

**FIGURE 2.**
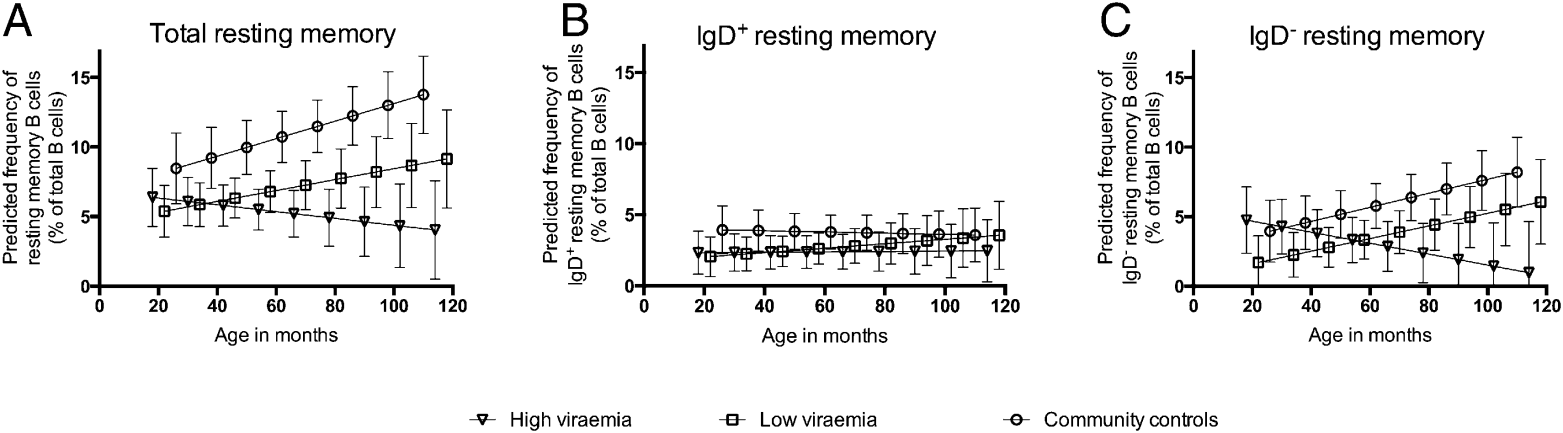
High HIV viremia interferes with age-related accumulation of IgD^−^ resting memory B cells. (**A**–**C**) Predicted proportions of total resting memory B cells, IgD^+^ resting memory B cells, and IgD^−^ resting memory B cells, respectively, after stratifying the HIV-infected children on the basis of their level of viremia. The PREDXCON STATA package was used to make quantile regression predictions with adjustments for HAART treatment and level of CD4^+^ T cell percentages.

In multivariable analyses, a generalized quantile regression model was used to assess the effect of age, HIV status, high viremia, low CD4^+^ T cell percentages, HAART, and an interaction term for age and high viremia on the proportions of each B cell subset. Notably, there were significant statistical interactions between high viremia and age with regard to proportions of total and switched, but not unswitched, resting memory B cells, suggesting that high viremia modified the age-dependent accumulation of switched resting memory B cells (Table II, Fig. 2). Similar interactions were observed on naive and activated mature B cells. As a result, additional multivariable quantile regression analyses were performed separately for high-viremia and low-viremia children (Table II).

**Table II.** Estimated change (β coefficients) in frequencies of various B cell subsets in relation to variations in age, HIV status, level of CD4^+^ T cell percentages, level of viremia, and HAART treatment status in multivariable quantile regression

	All Children		CC and HV		CC and LV	
	β Coefficient (SE)	*p* Value	β Coefficient (SE)	*p* Value	β Coefficient (SE)	*p* Value
Naive						
Age	−0.5 (0.8)	0.562	1.2 (0.7)	0.117	−0.1 (0.7)	0.850
HIV status	−4.7 (5.1)	0.354	**-21.6 (4.9)**	**<0.0005**	5.0 (5.7)	0.390
High viremia	−**30.8 (6.9)**	**<0.0005**				
Low CD4%	−7.2 (3.8)	0.059	**-10.4 (4.8)**	**0.035**	**-15.8 (4.9)**	**0.002**
HAART	**13.8 (4.0)**	**0.001**	**16.3 (5.0)**	**0.002**	3.9 (5.3)	0.466
Age*high viremia	**3.0 (1.4)**	**0.038**				
Resting memory						
Age	**0.5 (0.2)**	**0.012**	−0.1 (0.2)	0.485	0.5 (0.3)	0.097
HIV status	−**3.6 (1.2)**	**0.003**	**-4.5 (1.4)**	**0.002**	−3.7 (2.4)	0.119
High viremia	2.3 (1.7)	0.166				
Low CD4%	−1.8 (0.9)	0.054	−2.2 (1.4)	0.117	−1.1 (2.0)	0.596
HAART	−0.2 (1.1)	0.812	−0.3 (1.4)	0.823	−0.2 (2.2)	0.927
Age*high viremia	−**0.8 (0.4)**	**0.027**				
Activated mature						
Age	0.1 (0.2)	0.740	−0.2 (0.3)	0.552	0.0 (0.1)	0.894
HIV status	**2.1 (1.0)**	**0.047**	**6.0 (1.7)**	**0.001**	−0.9 (1.2)	0.438
High viremia	**11.7 (1.4)**	**<0.0005**				
Low CD4%	0.1 (0.8)	0.912	1.0 (1.7)	0.565	1.3 (1.0)	0.190
HAART	−**3.9 (0.8)**	**<0.0005**	−**4.8 (1.7)**	**0.008**	−0.6 (1.1)	0.552
Age*high viremia	−**1.5 (0.3)**	**<0.0005**				
Atypical memory						
Age	0.1 (0.6)	0.825	−0.9 (0.6)	0.159	0.1 (0.5)	0.791
HIV status	**5.2 (4.1)**	**0.031**	7.7 (4.0)	0.057	5.3 (4.3)	0.229
High viremia	**13.3 (5.7)**	**0.021**				
Low CD4%	5.9 (3.1)	0.057	**12.2 (4.0)**	**0.003**	5.9 (3.7)	0.115
HAART	−**7.2 (3.3)**	**0.031**	−6.2 (4.2)	0.142	−6.1 (4.0)	0.113
Age*high viremia	−0.6 (1.2)	0.614				
Plasmablasts						
Age	0.1 (0.2)	0.507	0.3 (0.2)	0.222	−0.0 (0.1)	0.879
HIV status	0.4 (1.1)	0.702	2.7 (1.5)	0.075	0.3 (1.1)	0.786
High viremia	2.0 (1.6)	0.202				
Low CD4%	−0.1 (0.8)	0.908	−0.5 (1.5)	0.736	0.7 (1.0)	0.459
HAART	−0.9 (0.9)	0.296	−1.2 (1.6)	0.464	−1.0 (1.1)	0.347
Age*high viremia	−0.1 (0.3)	0.786				
Immature/transitional						
Age	−0.3 (0.3)	0.277	−0.1 (0.3)	0.770	−0.4 (0.4)	0.268
HIV status	0.5 (1.9)	0.805	3.3 (2.1)	0.134	−0.5 (3.3)	0.876
High viremia	−0.2 (2.6)	0.953				
Low CD4%	2.0 (1.4)	0.153	−**4.4 (2.1)**	**0.045**	**6.6 (2.8)**	**0.020**
HAART	−2.5 (1.5)	0.099	0.2 (2.2)	0.928	−1.9 (3.0)	0.533
Age*high viremia	0.3 (0.5)	0.554				
IgD^+^ resting memory						
Age	0.2 (0.1)	0.111	−0.0 (0.1)	0.700	0.1 (0.1)	0.456
HIV status	−1.3 (0.7)	0.092	−1.6 (0.8)	0.057	−**2.1 (1.0)**	**0.045**
High viremia	0.6 (1.1)	0.620				
Low CD4%	−1.0 (0.6)	0.098	−0.5 (0.9)	0.549	−0.3 (1.0)	0.751
HAART	0.0 (0.6)	0.958	−0.6 (0.9)	0.478	1.0 (1.0)	0.320
Age*high viremia	−0.2 (0.2)	0.457				
IgD^−^ resting memory						
Age	**0.5 (0.2)**	**0.001**	−0.0 (0.2)	0.905	**0.5 (0.2)**	**0.006**
HIV status	−**2.3 (1.1)**	**0.035**	−**2.7 (1.2)**	**0.030**	−2.4 (1.7)	0.165
High viremia	**4.8 (1.6)**	**0.004**				
Low CD4%	−1.6 (0.9)	0.069	−2.1 (1.3)	0.123	−1.6 (1.6)	0.316
HAART	−0.1 (0.9)	0.891	0.7 (1.4)	0.615	−0.1 (1.6)	0.939
Age*high viremia	−**1.0 (0.3)**	**0.001**				

Age*high viremia is the interaction term for age and level of viremia. Every B cell subset was independently run in a quantile regression model containing age, HIV status, level of viremia, level of CD4^+^ T cell percentages, HAART treatment, and age*high viremia. Because there were statistical interactions between age and level of viremia, additional analyses were performed separately for high-viremia and low-viremia groups.

The *p* values < 0.05 were considered significant. Significant results are in bold text.

CC, community controls; HV, high-viremia group; LV, low-viremia group.

Among the high-viremia children, HIV infection significantly predicted high proportions of activated mature B cells, low proportions of naive B cells, and low proportions of both total and class-switched resting memory B cells. Low CD4^+^ T cell percentage significantly predicted low proportions of naive B cells, high proportions of tissue-like memory B cells, and low proportions of immature/transitional B cells, whereas being HAART naive significantly predicted low proportions of naive B cells and high proportions of activated mature B cells. These data are in agreement with previous studies in adults, suggesting that defects in B cell subset distribution are similar for highly viremic adults and children (7, 8) (Table II).

Among the low-viremia children, HIV infection predicted only low proportions of unswitched resting memory B cells, suggesting minimal effect of HIV on other B cell subsets in the absence of high viremia or recovery of the B cell compartment when viremia is controlled by HAART. Switched resting memory B cells were acquired with age, although at a lower rate than in healthy community controls (Fig. 2C). Low CD4^+^ T cell percentage was a significant predictor of low proportions of naive B cells and, interestingly, high proportions of immature/transitional B cells, a finding that has been reported elsewhere (20) (Table II).

### The frequencies of Ag-specific memory B cells are reduced in HIV-infected children

Upon continued recruitment of study participants, additional children were involved in the second phase of the study to allow analysis of Ag-specific B cell responses, bringing the number of HIV-infected children to 116 (52 high viremia; 64 low viremia) and community controls to 58 (Table III). Children were omitted from the analyses of TT and pneumococcal responses if they had received a pneumococcal conjugate vaccine that contained TT as a conjugating protein in a community catch-up campaign that followed the rolling out of the pneumococcal vaccine during the study period. Those without samples or the respective childhood vaccination records were also omitted. The numbers of samples analyzed for each Ag are shown in Supplemental Table II.

**Table III.** Characteristics of the children whose samples were analyzed for function of B cells

	High Viremia		Low Viremia		Community Controls
	Value	*p* Value*^a^*	Value	*p* Value*^a^*	
*n*	52		64		58
Age (y)	4.1 (2.8–6.0)	0.158	4.8 (2.5–7.2)	0.5623	4.6 (3.6–6.8)
% Female (*n*)	46 (24)	0.824	56 (35)	0.423	48 (28)
% on HAART (*n*)	40(21)	N/A	78(50)	N/A	N/A
Viral load*^b^*	4.7 (4.4–5.2)	N/A	1.4 (0.0–3.2)	N/A	N/A
% CD4^+^ T cells*^c^*	21.3 (9.4–27.4)	<0.0005	29.4 (22.5–34.5)	0.007	33.8 (28.1–39.3)
% CD8^+^ T cells*^c^*	37.2 (30.9–47.3)	<0.0005	29.0 (24.4–37.5)	<0.0005	15.8 (13.7–17.0)
% B cells*^c^*	17.8 (9.8–22.2)	0.061	15.3 (11.2–20.7)	0.009	20.5 (15.7–23.7)

Values shown are medians, 25th and 75th percentiles, unless otherwise stated. Statistical test: Wilcoxon rank sum test, except for % female, where χ^2^ test was used.

^*a*^ The *p* values correspond to comparison between the community controls and either high-viremia or low-viremia HIV-infected groups.

^*b*^ Viral load is in log_10_ RNA copies per milliliter.

^*c*^ Percentage of total lymphocytes.

N/A, not applicable.

**FIGURE 3.**
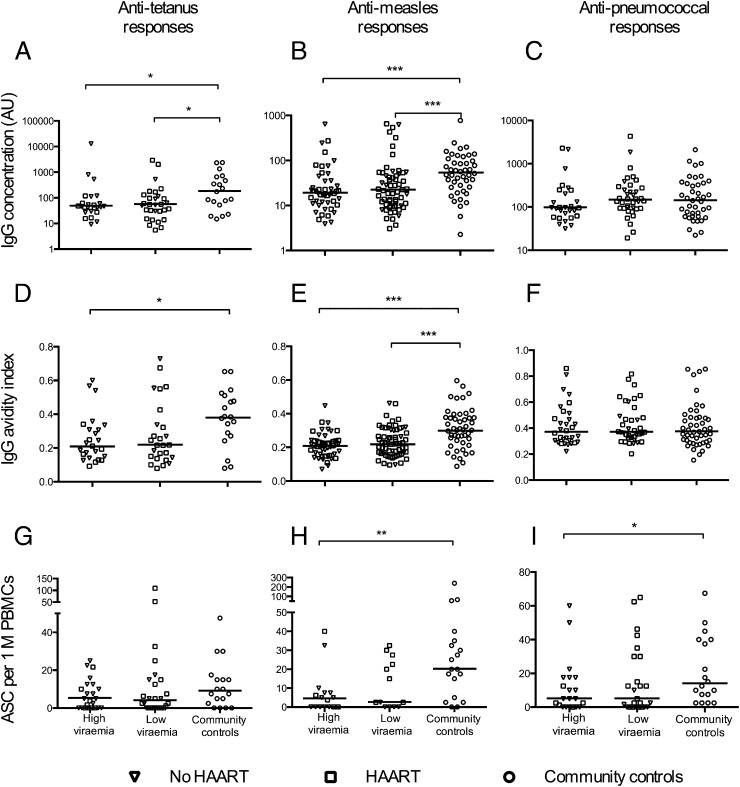
HIV infection in children is associated with poor B cell responses against some childhood vaccine Ags and infections. (**A**–**I**) Comparison of the B cell responses [plasma Abs (A–C), IgG avidities (D–F), and memory B cells (G–I)] against TT, measles Ag, and pneumococcal capsular polysaccharides, respectively, between the study groups after stratifying the HIV-infected children on the basis of their level of viremia. Each symbol represents an individual child. Horizontal lines represent medians. High-viremia group, ≥5000 RNA copies per milliliter; low-viremia group, <5000 RNA copies per milliliter. Wilcoxon rank-sum test. *p < 0.05, **p < 0.01, ***p < 0.001.

HIV-infected children showed lower plasma concentrations and avidities of IgG against TT and measles, but not against pneumococcal polysaccharides, independent of viremia when compared with community controls, although anti-TT IgG avidities in HIV-infected children with low viremia did not reach statistical significance. The frequencies of memory B cells to measles and pneumococcal polysaccharides were low in children with high viremia compared with community controls, whereas they tended to be lower but not significantly different in children with low viremia (Fig. 3). Notably, HAART-treated children had higher frequencies of memory B cells against measles Ag and pneumococcal capsular polysaccharides than the HAART-naive children within the low-viremia group (*p* = 0.0046 and 0.0162, respectively), probably because HAART-naive children were younger and had received less environmental exposure. Adjusting for multiple comparisons (Bonferroni adjustment) did not change the interpretation of data on any response against measles and avidity of IgG against TT. However, quantities of IgG against TT and frequencies of memory B cells against pneumococcal polysaccharides were no longer different between the different groups of children after a Bonferroni adjustment.

Direct correlations were observed between frequencies of Ag-specific memory B cells (as determined in cultured B cell ELISPOT) and frequencies of resting memory B cells (as determined by flow cytometry), with the exception of anti-measles memory B cells that correlated with only unswitched resting memory B cells, suggesting that the different Ag-specific responses contributed to the observations made on resting memory B cell pools (Supplemental Table III).

### The expression of BAFF receptors on B cells and plasma levels of BAFF are altered in HIV-infected children

BAFF is a B cell survival factor whose altered receptor expression has been associated with decreased B cell survival in HIV-infected adults (11). Measurements were done on plasma BAFF levels and the expression of its receptors, BAFF receptor (BAFF-R/BR3) and TACI, on B cells.

**FIGURE 4.**
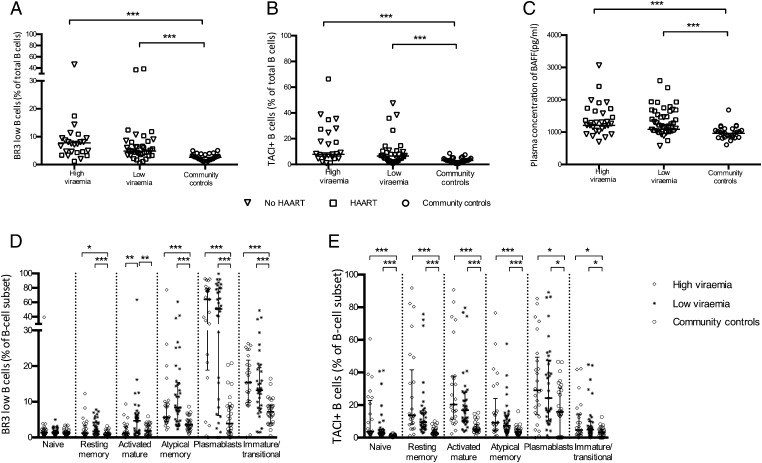
HIV infection in children is associated with altered expression of BAFF receptors (increased frequencies of BR3 low and TACI^+^ B cells) and increased plasma BAFF levels. (**A**–**C**) Comparison of frequencies of BR3^low^ B cells, frequencies of TACI^+^ B cells, and plasma BAFF levels, respectively, between the study groups. (**D** and **E**) Comparison of expression of BR3 and TACI, respectively, in various subsets of B cells between the study groups. HIV-infected children were stratified on the basis of their level of viremia. Each symbol represents an individual child. Horizontal lines represent medians. High-viremia group, ≥5000 RNA copies per milliliter; low-viremia group, <5000 RNA copies per milliliter. Wilcoxon rank-sum test. *p < 0.05, **p < 0.01, ***p < 0.001.

The proportions of B cells with low expression of the BR3 (BR3^low^) were higher in HIV-infected children, independent of viremia, when compared with community controls (Fig. 4A). In multivariable analyses in a quantile regression model, high viremia was a predictor (*p* = 0.016) of high proportions of BR3^low^ B cells (Table IV). As expected, the proportion of BR3^low^ cells was highest for plasmablasts and immature/transitional B cells when the expression of BR3 was evaluated in healthy children in the different B cell subsets but significantly increased on these subsets in HIV-infected children (Fig. 4D). In addition, the proportion of BR3^low^ atypical memory B cells was increased in HIV-infected children independent of viremia, as had been reported for adults infected with HIV. However, we also observed increased proportions of BR3^low^ resting memory B cells as well as activated B cells in HIV-infected children and those with low viremia, respectively, which may render these cells more susceptible to apoptosis. Together, our data suggest that HIV infection could, by itself, be associated with altered expression levels of BR3 even within a B cell subset (Fig. 4D).

**Table IV.** Estimated change (β coefficients) in frequencies of BR3^low^ and TACI^+^ B cells in relation to variations in age, HIV status, level of CD4^+^ T cell percentages, level of viremia, HAART treatment status, and an interaction term for age and level of viremia

	β Coefficient (SE)	*p* Value
BR3^low^		
Age	−0.1 (0.2)	0.631
HIV status	1.8 (1.3)	0.169
High viremia	**4.6 (1.9)**	**0.016**
Low CD4%	1.3 (1.0)	0.207
HAART	−0.4 (1.1)	0.724
Age*high viremia	**-0.8 (0.4)**	**0.035**
TACI^+^		
Age	−0.0 (0.5)	0.986
HIV status	1.5 (3.2)	0.635
High viremia	−7.8 (4.7)	0.102
Low CD4%	**6.2 (2.6)**	**0.018**
HAART	0.2 (2.7)	0.937
Age*high viremia	**2.3 (1.0)**	**0.025**
Plasma BAFF (pg/ml)		
Age	17.4 (23.7)	0.464
HIV status	102.6 (162.4)	0.529
High viremia	109.8 (259.7)	0.673
Low CD4%	155.2 (119.3)	0.197
HAART	**399.6 (132.5)**	**0.003**
Age*high viremia	−0.3 (4.6)	0.945

Age*high viremia is the interaction term for age and level of viremia. The frequency of BR3^low^ B cells, TACI^+^ B cells, and plasma concentrations of soluble BAFF were independently run in a quantile regression model containing age, HIV status, level of viremia, level of CD4^+^ T cell percentages, HAART treatment and age*high viremia.

The *p* values < 0.05 were considered significant. Significant results are in bold text.

The proportions of B cells expressing TACI (TACI^+^) were also higher in HIV-infected children, independent of viremia, when compared with community controls (Fig. 4B). In multivariable analyses in a quantile regression model, low CD4^+^ T cell percentages significantly predicted high proportions of TACI^+^ B cells (*p* = 0.018) (Table IV). The expression of TACI on B cell subsets among HIV-infected children was highly variable but significantly increased on all B cell subsets, compared with healthy children (Fig. 4E).

The plasma concentrations of soluble BAFF were also elevated in the HIV-infected children, notably in the HAART-treated children, that is, in the high-viremia group (HAART naive; median 1207.4 pg/ml [IQR 976.6–1305.7] versus HAART treated; median 1689.6 pg/ml [IQR 1390.5–1865.7], *p* = 0.0047) and in the low-viremia group (HAART naive; median 1088.0 pg/ml [IQR 943.7–1302.4] versus HAART treated; median 1433.9 pg/ml [IQR 1165.7–1734.6], *p* = 0.0107) (Fig. 4C). In multivariable analyses in a generalized quantile regression, treatment with HAART was the sole predictor of high levels of plasma BAFF (Table IV).

Correlations analyses revealed inverse correlations between BAFF and total B cell percentages (as proportion of lymphocytes). BAFF levels also correlated inversely with proportions of total resting memory B cells, IgD^+^ resting memory B cells, and IgD^−^ resting memory B cells when all children were considered, but not after stratifying them on the basis of HIV status and viremia level (Table V). There were no correlations between Ag-specific responses and BAFF levels after adjusting for multiple comparisons (data not shown).

**Table V.** Spearman correlation coefficients rho (*p* values) between frequencies of B cell subsets and plasma concentrations of BAFF

	All Participants Rho (*p*)	HIV-Infected High-Viremia Rho (*p*)	HIV-Infected Low-Viremia Rho (*p*)	Community Controls Rho (*p*)
B cell subset				
Naive	−0.09 (n/s)	−0.42 (n/s)	0.19 (n/s)	−0.06 (n/s)
Resting memory	**−0.41 (0.006)**	−0.22 (n/s)	−0.24 (n/s)	−0.17 (n/s)
Activated mature	−0.04 (n/s)	−0.01 (n/s)	−0.33 (n/s)	−0.15 (n/s)
Atypical memory	0.21 (n/s)	0.36 (n/s)	−0.15 (n/s)	0.04 (n/s)
Plasmablasts	0.01 (n/s)	−0.07 (n/s)	−0.16 (n/s)	0.15 (n/s)
Immature/transitional	−0.13 (n/s)	−0.36 (n/s)	−0.07 (n/s)	0.13 (n/s)
IgD^+^ resting memory	**−0.38 (0.026)**	0.01 (n/s)	−0.32 (n/s)	−0.02 (n/s)
IgD^−^ resting memory	**−0.40 (0.013)**	−0.26 (n/s)	−0.22 (n/s)	−0.06 (n/s)

All children were evaluated together, and then subsequent correlations were restricted to each group. Bonferroni corrections were done on all *p* values.

Corrected *p* values < 0.05 were considered significant. Statistically significant correlations are in bold text.

n/s, not significant.

## Discussion

Despite the gains in prevention of mother-to-child transmission, many children still become infected vertically; ∼260,000 children acquired HIV infection in 2012 in low- and middle-income countries. Furthermore, only 34% of eligible children were receiving HAART in 2012 (21). If left untreated, most HIV-infected children die within 2 y, probably owing to their inability to launch adequate immune responses (22). A better understanding of their immunological defects could help to design better interventions to enhance their chance of survival. Detailed descriptions of the B cell defects in adults infected with HIV have been reported (7, 8, 23–25), but descriptions in children to the same depth are lacking.

In this study we observed that HIV-infected children, similar to adults, had an expansion of activated B cell subsets (23, 25). In this study, we analyzed activated B cell subsets in more detail, compared with a previous study in children (26). Activated mature B cells, tissue-like memory B cells, and plasmablasts were expanded in HIV-infected children in a viral load–dependent manner, with additional contributions from low CD4^+^ T cell percentages, suggesting that viral factors could be directly activating B cells. HIV gp120, CD40L on HIV virions, ferritin from macrophages in response to HIV Nef, inflammatory B cell cytokines and bystander effect of activated T cells could all contribute to this activation (27–31).

In agreement with other studies, we observed a reduction of circulating resting memory B cells (26, 32, 33). Of interest, there was statistical interaction between age and high viremia with regard to accumulation of resting memory B cells. Children are expected to acquire memory B cells with age owing to the continued exposure to Ags. Indeed, community controls and HIV-infected children with low viremia showed an increase in proportion of switched resting memory B cells with age, whereas HIV-infected children with high viremia showed an age-dependent decrease. HIV-infected adults who have high viremia have been previously reported to generate poor Ab responses against vaccines, suggesting that the highly viremic children in our cohort could have been unable to generate high frequencies of memory B cells (34). However, HIV infection also depletes pre-existing memory B cells in adults, and high viremia could have depleted the few memory B cells that were generated in these children, especially among the older ones (9). In addition, sequestration of memory B cells in tissues cannot be ruled out because B cells from HIV patients have increased chemotactic response to chemokines that direct them to lymphoid tissues (35, 36). Notably, proportions of resting memory B cells in the low-viremia group still remained consistently lower than in community controls, probably because of prophylaxis with cotrimoxazole that could have reduced overall exposure to bacterial Ags and malaria. Alternatively, the remaining effects of HIV on T cell function, even in the absence of high viremia, may reduce T cell help and hence affect the generation of memory B cells.

Similar to previous studies in children, HIV infection was associated with lower IgG levels, IgG avidities, and frequencies of memory B cells against childhood vaccines (32, 37–39). The low responses were observed in both high- and low-viremia groups, suggesting that spontaneous recovery did not occur after controlling viremia, and re-exposure to the Ag (e.g., revaccination) may be necessary to rebuild Ag-specific B cell memory once viremia is controlled. HAART-treated adults make better Ab responses to vaccines when compared with their HAART-naive HIV-infected counterparts (34, 40). Of note, HIV spared the responses against pneumococcal capsular polysaccharides with the exception of frequencies of memory B cells. Despite cotrimoxazole prophylaxis, interaction between pneumococcal exposure and HIV infection cannot be ruled out; HIV-infected children could be exposed to more pneumococcal infections owing to their impaired immune function or to the presence of sick HIV-infected parents in the household, and such increased exposure could lead to increased Ab responses that mask the deleterious effects of HIV (41).

Because HIV could be affecting the B cell compartment by modulating the levels of plasma cytokines and expression of their receptors on B cells, we assessed the effect of BAFF, a B cell cytokine that is strongly implicated in B cell immunopathology in other diseases and whose profile is altered in HIV-infected adults (42–44). We observed altered expression of BAFF receptors and increased plasma BAFF levels in HIV-infected children, similar to previous reports in adults (10, 11). We also noted intersubset variations in the expression of BAFF receptors, a phenomenon associated with B cell developmental stages and activation status (45, 46). When coupled with altered frequencies of the B cell subsets, the intersubset variations could contribute to the overall alterations in expression of BAFF receptors. In contrast, frequencies of BR3^low^ B cells within each B cell subset were higher in the HIV-infected groups, suggesting that HIV also caused downregulation of BR3 in B cells independently of altering B cell subset distribution. Low expression of BR3 could render the B cells incapable of harnessing survival signals from BAFF, leading to B cell depletion. Alternatively, high plasma BAFF levels have been implicated in downregulating BR3 expression, either by mediating internalization or by shedding (42, 43, 46). Activation of B cells has been shown to upregulate expression of TACI and could partly explain the increased frequencies of TACI^+^ B cells in the HIV-infected groups (45). Considering that BAFF has been implicated in pathological activation of B cells and maintenance of defective subsets in autoimmune diseases, it is possible that similar effects occur in HIV infection. However, BAFF levels did not correlate with the sizes of activated B cell subsets in our cohort. Notably, we observed an inverse correlation between plasma BAFF and resting memory B cell subsets. BAFF has been reported to synergize with CXCL13, a B cell chemokine that mediates movement of B cells into follicles and whose plasma levels have been shown to be elevated in HIV infection. The synergy is particularly pronounced in memory B cells (36, 47). Thus, BAFF-mediated sequestration of memory B cells in the tissues of HIV patients, similar to that suspected to occur in some autoimmune conditions, cannot be ruled out (48). BAFF-driven terminal differentiation of memory B cells could also account for the lower frequencies of memory B cells (49). Alternatively, the relationship between BAFF and resting memory B cells could be driven by the altered BR3 expression that could in turn be responsible for poor survival of the memory B cells (11).

Surprisingly, higher BAFF levels were observed in HAART-treated children. At the time of the study, children in this age group were placed on HAART only if they had clinically or immunologically deteriorated (12). Immune activation by HIV, which is associated with increased BAFF production, has been shown to predict the rate of disease progression in humans and primate models and could explain the elevated BAFF levels in children who had been placed on HAART because of historical immunological or clinical deterioration (50, 51). The persistence of elevated BAFF levels despite control of viremia in some children could be an indication of predisposition to elevated levels of proinflammatory cytokines in HIV and hence susceptibility to faster progression.

Thus, HIV-infected children show elevated plasma BAFF levels and altered BAFF receptor expression on their B cells, factors that could affect the survival, tissue distribution, and differentiation patterns of B cells. It will be important to determine the contribution of each of these effects in future studies.

In 2013, WHO conditionally recommended that all 2- to 5-y-old HIV-infected children be placed on HAART in a bid to simplify the guidelines so as to increase HAART coverage in resource-poor settings where immunological monitoring might be unavailable (52). Our results provide biological evidence of the benefits of early control of viremia in all HIV-infected children and emphasize the need to implement the new guidelines rigorously. Additional efforts to increase HAART coverage would enable more children to grow immunologically in a similar manner to healthy children.

## Supplementary Material

Data Supplement
