# Supplementary material for: Control of Viremia Enables Acquisition of Resting Memory B Cells with Age and Normalization of Activated B Cell Phenotypes in HIV-Infected Children
Source: J Immunol. 2015 Jun 26;195(3):1082–91. doi: 10.4049/jimmunol.1500491 (PMC4505960; doi:10.4049/jimmunol.1500491)
Supplement: Data Supplement [file JI_1500491.zip › JI_1500491_Supplemental_Material_1.pdf]

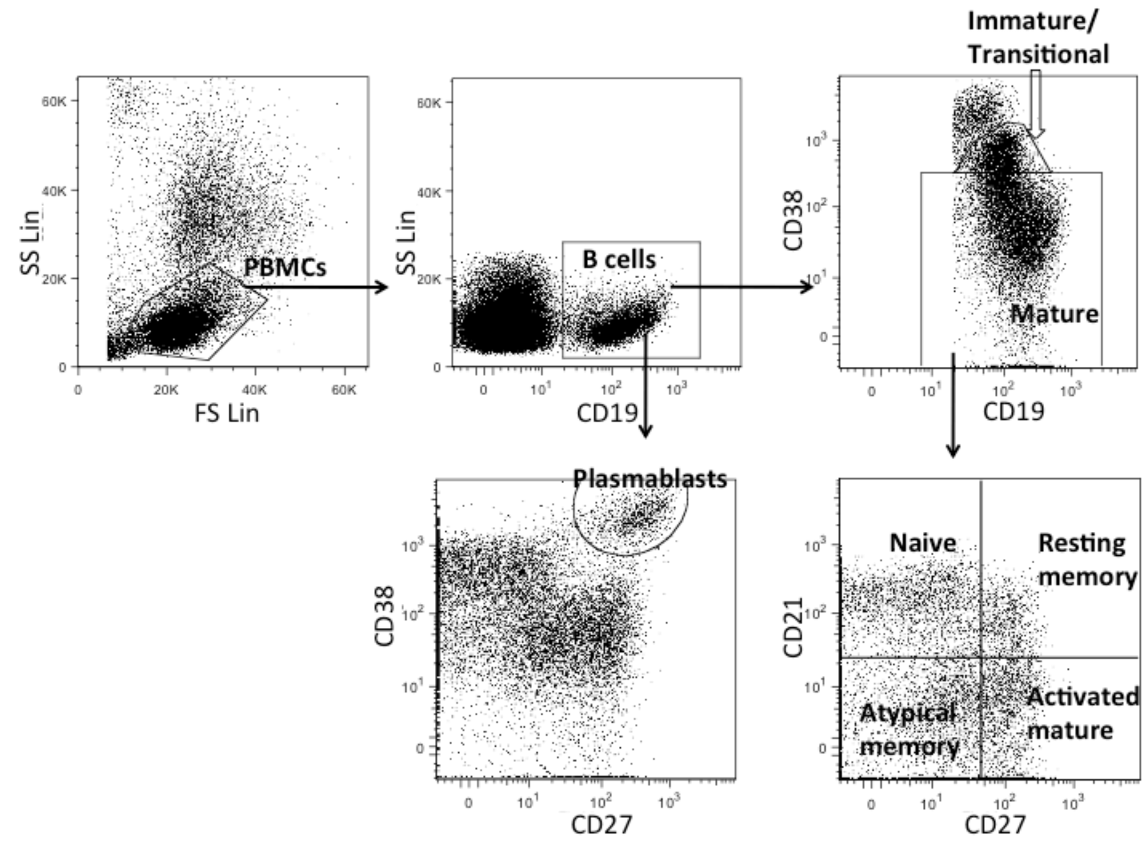

Supplementary figure 1: Gating strategy for determining the different B-cell subsets was done as follows: Immature/transitional, $CD19^{+}CD10^{+}CD38^{++}CD27^{-}$; naive, $CD19^{+}CD27^{-}CD21^{+}$; tissue-like memory, $CD19^{+}CD27^{-}CD21^{-}$; resting memory, $CD19^{+}CD27^{+}CD21^{+}$; activated mature, $CD19^{+}CD27^{+}CD21^{-}$; Plasmablasts, $CD19^{+}CD27^{++}CD38^{+++}$; unswitched resting memory, $CD19^{+}CD21^{+}CD27^{+}IgD^{+}$ and switched resting memory, $CD19^{+}CD21^{+}CD27^{+}IgD^{-}$.

Supplementary table I: Characteristics of the HAART-treated and HAART-naive HIV-infected children whose samples were analyzed for phenotypes of B cells.

	**High viraemia**			**Low viraemia**		
	No HAART	HAART	P value[a]	No HAART	HAART	P value[a]
N	25	11		13	29	
Age (years)	3.4 (2.5-5.0)	4.4 (3.0-6.2)	0.511	2.1 (1.6-2.6)	4.9 (3.4-7.0)	0.001
% female (n)	52(113)	36(4)	0.387	46(6)	64(18)	0.273
Viral load [b]	4.9 (4.3-5.4)	4.8 (4.4-5.0)	0.415	2.5 (0.0-3.3)	0.7 (0.0-3.0)	0.376
% $CD4^+$ T-cells [c]	18.9 (8.8-27.4)	23.1 (13.0-26.6)	0.927	23.0 (20.4-33.4)	29.8 (25.5-36.3)	0.259
% $CD8^+$ T-cells [c]	37.2 (31.8-43.9)	35.4 (29.4-50.4)	0.868	25.5 (16.6-38.4)	29.4 (24.5-40.7)	0.259
% B-cells [c]	17.9 (11.8-24.4)	16.9 (9.3-19.1)	0.179	15.5 (11.0-23.5)	15.0 (11.8-19.5)	0.881

Values shown are medians (inter-quartile range) unless otherwise stated.

[a] P values correspond to comparison between the HAART-naïve and HAART-treated subgroups within either the high viraemia or low viraemia groups.

[b] Viral load is in log_{10} RNA copies/ml.

[c] percentage of total lymphocytes.

Statistical test; Wilcoxon rank sum test, except for % female where chi squared test was used.

Supplementary table II: Number of children whose samples were analyzed for various parameters

	**High viraemia**	**Low viraemia**	**Community controls**
N	52	64	58
B-cell subsets	36	42	28
Anti-TT IgG concentration and	31	38	19
Anti-PPS IgG concentration and	28	38	41
Anti-measles IgG concentration	49	61	45
Anti-TT IgG ASC (B-cell	29	33	18
Anti-PPS IgG ASC (B-cell	21	25	18
Anti-measles IgG ASC (B-cell	16	13	19

Supplementary table III: Spearman's correlation coefficients rho (P values) between frequencies of resting memory B cells and measures of function of B cells.

	Resting memory	IgD^- resting memory	IgD^+ resting memory
**Anti-TT IgG ASC/1MPBMCs**	**0.5942 (0.0022)**	**0.5177 (0.0187)**	**0.6591 (<0.00005)**
**Anti-Measles IgG ASC/1MPBMCs**	0.4229 (0.1397)	0.3911 (0.2442)	**0.5515 (0.0077)**
**Anti-PPS IgG ASC/1MPBMCs**	**0.5817 (0.0033)**	**0.5060 (0.0253)**	**0.6196 (0.0011)**

Spearman’s correlation with Bonferroni adjustments were done. P values below 0.05 were considered as significant. Statistically significant correlations are in bold text.
